# Targeted Chromatinization and Repression of HIV-1 Provirus Transcription with Repurposed CRISPR/Cas9

**DOI:** 10.3390/v12101154

**Published:** 2020-10-12

**Authors:** Alex Olson, Binita Basukala, Seunghee Lee, Matthew Gagne, Wilson W. Wong, Andrew J. Henderson

**Affiliations:** 1 Department of Medicine and Microbiology, Boston University School of Medicine, Boston, MA 02118, USA; alex.olson@bmc.org (A.O.); Matthew.Gagne@nih.gov (M.G.); 2 Department of Biology, Boston University, Boston, MA 02215, USA; binitab@bu.edu; 3 Biomedical Engineering and Biological Design Center, Boston University, Boston, MA 02215, USA; slee02@bu.edu (S.L.); wilwong@bu.edu (W.W.W.)

**Keywords:** HIV transcription, HIV latency, CRISPR, chromatin

## Abstract

The major barrier to HIV-1 cure is the persistence of latent provirus, which is not eradicated by antiretroviral therapy. The “shock and kill” approach entails stimulating viral production with latency-reversing agents followed by the killing of cells actively producing the virus by immune clearance. However, this approach does not induce all intact proviruses, leaving a residual reservoir. CRISPR/Cas9 has been utilized to excise integrated Human Immunodeficiency Virus (HIV) DNA from infected cells in an RNA-guided, sequence-specific manner. Here, we seek to epigenetically silence the proviral DNA by introducing nuclease-deficient disabled Cas9 (dCas9) coupled with a transcriptional repressor domain derived from Kruppel-associated box (KRAB). We show that specific guide RNAs (gRNAs) and dCas9-KRAB repress HIV-1 transcription and reactivation of latent HIV-1 provirus. This repression is correlated with chromatin changes, including decreased H3 histone acetylation and increased histone H3 lysine 9 trimethylation, histone marks that are associated with transcriptional repression. dCas9-KRAB-mediated inhibition of HIV-1 transcription suggests that CRISPR can be engineered as a tool for block-and-lock strategies.

## 1. Introduction

Human immunodeficiency virus type 1 (HIV-1) is the causative agent of acquired immunodeficiency syndrome (AIDS). The primary targets of HIV-1 are CD4+ T cells, which are diminished by HIV-1 infection, directly contributing to immunodeficiency. Current antiretroviral therapy (ART) halts viral replication and disease progression. However, discontinuation of ART leads to rapid rebound of HIV-1 replication, revealing that ART does not target persistent latent HIV-1 infection. This latent reservoir, with an estimated half-life of 44 months [1,2], has been shown to be established early at the time of infection and/or at the time of ART initiation [3]. Resting CD4+ T cells and other long-lived memory CD4+ T cell populations are posited to harbor the bulk of latent HIV infection [1,4,5]. This reservoir is established as a result of direct infection of quiescent naive or memory CD4+ T cells [6,7,8]; infection of CD4+ T cells differentially activated [9,10,11]; and the transition of infected, activated CD4+ T cells to a resting memory phenotype [12,13,14,15]. Multiple mechanisms, including lack of transcriptional activators, abundance of transcriptional repressors, stochastic fluctuation of Tat, and epigenetic changes cooperate to silence HIV-1 transcription to establish and maintain proviral latency. Latency presents the primary barrier to curing HIV infection.

Several strategies have been explored for eradication of latent HIV-1 reservoirs. For example, “shock and kill” strategies aim to reduce the reservoir with compounds that induce HIV-1 expression so that latent cells are cleared by anti-HIV immunity or HIV-1 mediated cell death [16,17,18,19]. Cytokines, protein kinase C agonists, histone deacetylases inhibitors, and SMAC mimetics are some examples of compounds that have been employed as latency-reversing agents, but, despite inducing modest HIV-1 transcription, they have had limited impact on decreasing the HIV-1 reservoir in in vivo models [17,19,20]. The lack of efficacy of latency-reversal agents suggests a need for alternative strategies that specifically target HIV-1 provirus [21,22,23].

With the development of versatile genome editing tools such as zinc finger nucleases, TAL effector nucleases, and RNA-guided CRISPR/Cas9 systems, it is possible to directly target and modify the HIV-1 genome [24,25,26]. Furthermore, these tools have been used to make cells resistant to HIV-1 infection by modifying HIV-1 receptors, CCR5, and/or CXCR4 [27,28,29,30]. In addition, CRISPR/Cas9-derived systems have been used to directly disrupt and excise integrated HIV-1 provirus from infected cells in vitro and in vivo [31,32,33,34,35]. Cas9 has also been engineered to modulate transcription by fusing nuclease-deficient Cas9 with transcriptional activator or repressor domains while preserving the Cas9 nucleic acid binding domain. The strategy of using repurposed CRISPR/Cas9 as a transcriptional activator has been explored as a tool to reverse HIV-1 latency [36,37], demonstrating that Cas9 provides therapeutic opportunities beyond gene editing modifications.

We employed a CRISPR/Cas9 system to repress and block induction of latent HIV-1. To silence HIV-1 transcription, we targeted the HIV-1 LTR using complementary guide RNA (gRNA) and enzymatically inactive Cas9 nuclease-derived protein disabled Cas9 (dCas9) fused to a repression domain derived from Kruppel-associated box (KRAB), which, through the recruitment of KAP1, promotes epigenetic silencing of retroviruses and endogenous retroelements to protect the integrity of the genome [38,39,40,41,42]. Our results provide a proof of concept that dCas9-KRAB induces epigenetic changes at targeted sites to repress HIV-1 transcription.

## 2. Materials and Methods

### 2.1. Cell Culture

HEK293T cells were maintained in DMEM (Corning 10017CV, Corning, NY, USA) containing 10% Fetal Bovine Serum (FBS; bio-techne S12450) and 100 U/mL Penicillin/Streptomycin (P/S; Gibco 15140163, Gaithersburg, MD, USA). Jurkat T cells and J-Lat 6.3 [43] cell lines were maintained in RPMI 1640 (Gibco 11875135, Gaithersburg, MD, USA) containing 10% heat-inactivated FBS, P/S. Cells were incubated at 37 °C, 5% CO_2_. All cell lines were obtained from ATCC (Manassas, VA, USA).

### 2.2. dCas9 Constructs

gRNA expression constructs were designed for HIV NL4-3 genome sequence using the online molecular biology software Benchling Software, 2018 (www.benchling.com). HIV-1 specific sequences were 21 bp in length, preceded by a protospacer adjacent motif (PAM) sequence “NGRRT” on the complementary strand, and followed by the Cas9 scaffolding region. Single gRNA sequences spanning the HIV genome were assigned specificity and activity scores on a scale of 01–00 using Benchling software. Specificity scores describe the off-target potential for gRNA binding compared to DNA libraries of interest [44,45,46]. The activity score predicts endonuclease activity of Cas9 at the target site, which is influenced by size and secondary structure of gRNA [44,45,46]. Guide sequences met a threshold specificity score greater than 50 (range 67.29–6.4), whereas there was a range of activity scores (range 3.16–6.4) (Table 1), suggesting that all guides are specific for the HIV genome but may not be optimally active. Sequences were either cloned into expression vectors as described below or obtained as Ultra RNA Oligos (Integrated DNA Technologies, Coralville, IA, USA).

The dCas9-KRAB construct was created by fusing dCas9 and KRAB domain through Gibson isothermal assembly. dCas9 was derived from *Staphylococcus aureus* (Addgene 68495, Watertown, MA, USA)[47], and the KRAB domain fragment was amplified using PCR from pEx1-pEF-H2B-mCherry-T2A-rTetR-KRAB-Zeo (Addgene 78352, Watertown, MA, USA)[48] and inserted into dCas9 construct. The gRNAs were cloned using a template plasmid produced by gBlocks from Integrated DNA Technologies, and the inserts were created by annealing customized oligos (Invitrogen, Carlsbad, CA, USA) and ligated into plasmid. Table 2 summarizes dCas9 reagents.

### 2.3. Lentivirus Packaging

Lentiviruses were generated in HEK293T cells by Polyethylenimine (PEI; Sigma Aldrich 408727, Natick, MA, USA) transfection. 24 h prior to transfection, 5 × 10^6^ cells were plated in a T75 tissue culture flask. For transfection, 15 mg of total plasmid DNA was combined with 45 mg of PEI in 1.2 mL serum-free DMEM solution and incubated for 30 min prior to addition to cells. Single round HIV-1 was generated by cotransfection of pNL43-Δ*env*-luciferase. After 48 h, viral supernatants were collected and syringe-filtered using 0.45 µm filter (Fisher SLHV033RS, Waltham, MA, USA) prior to storage at −80 °C. Viruses were titrated on CEM cells containing a HIV-1 Tat driven GFP reporter and measured by flow cytometry. The multiplicity of infection (MOI) was estimated by multiplying percent of GFP-positive cells by the total number of cells infected, and divided by volume of inoculum.

### 2.4. Transductions and Infections

Three million cells were transduced or infected with lentiviruses (MOI 0.1) by spinoculation (60 min, 1200× *g*) in a six-well tissue culture plate with 1 mL of media. Cells were washed with PBS and resuspended in the media. Jurkat T cell and J-Lat 6.3 cell lines stably expressing dCas9-KRAB were generated by transducing cells with a dCas9-KRAB lentivirus containing blasticidin resistance and selected in RPMI complete media containing 10 µg/mL blasticidin (InvivoGen ant-bl-1, San Diego, CA, USA).

### 2.5. HEK293T Transfections

HEK293T cells were plated 24 h prior to being transfected using a 4:1 molecular weight ratio of PEI to plasmid DNA. For 96-well assay, 10,000 cells were plated and transfected with 125 ng of total DNA. For gRNA screens, equal parts of dCas9-KRAB, gRNA, and HIV NL43-Denv-Luciferase plasmids were used. pCDN3.1 vector was a control for DNA input. After 48 h, transfected cells were lysed for luciferase assay using Bright-Glo (Promega E2610, Madison, WI, USA). Cell viability was monitored with CellTiter Glo (Promega C7570, Madison, WI, USA). Since Bright-Glo and CellTiter-Glo are directly proportional, interassay variability was adjusted by normalizing to cell input.

### 2.6. JLat Reactivation

JLat 6.3 stably expressing dCas9-KRAB was removed from media containing blasticidin and split at 200,000 cells/mL 2 days prior to nucleofection. Cells were electroporated per manufacturer protocol (Lonza #VACA-1003, Portsmouth, NH, USA) with specifications and minor modifications as described below. Three hundred nM of Ultramer RNA Oligos gRNA constructs were used per condition using program X-005 on Nucleofector 2b Device (Lonza, Portsmouth, NH, USA). Following electroporation, 500 mL of warmed RPMI complete with 20% FBS was added to the cuvette and allowed to rest for 10 min prior to being transferred to a 12-well tissue culture plate in total volume of 1.5 mL overnight. Cells were washed with PBS and resuspended in RPMI complete with 10% FBS for 4 days. On day 5, 100,000–200,000 cells were stimulated in duplicate with PMA (10 mg/mL) and ionomycin (2.5 nM) for 18 h in 96 well-plate. Conditions were pooled and harvested for flow cytometry or qPCR. Percentage of reactivation was determined by GFP expression compared to a scramble control. RNA expression was determined using qPCR targeting HIV-GFP reporter transcription to avoid any lentiviral interference. HIV-1 RNA expression was normalized to GAPDH expression.

### 2.7. HIV-1 RNA and DNA Quantification

RNA and DNA from HIV-1 infected cells were isolated by All Prep DNA/RNA kits (Qiagen 80204, Germantown, MD, USA) or Trizol (Invitrogen 15596026) per manufacturer protocol, and RT-qPCR was performed on extracted and purified RNA using 4X Fast Virus Master Mix (Applied Biosystems 4444434, Beverly, MA, USA) targeting HIV-1 5′ LTR region (forward 5′-TACTGACGCTCTCGCACC-3′; reverse 5′- TCTCGACGCAGGACTCG-3′; probe 5′-FAM-CTCTCTCCTTCTAGCCTC-3′) or GFP (forward 5′-CTGACCCTGAAGTTCATCTG-3′; reverse 5′-GAAGTCGTGCTGCTTCAT-3′; probe 1X SYBR green). HIV-1 expression was normalized to GAPDH (forward 5′-TGATGACATCAAGAAGGTGGTGAAG-3′; reverse 5′-TCCTTGGAGGCCATGTGGGCCAT-3; Probe 1X SYBR green). HIV-1 RNA was quantified by HIV-1 RNA standard curve ranging from 10 to 1,000,000 copies. PCR conditions were 5 min at 50 °C, 40 cycles of 15 s at 95 °C, and 30 s at 60 °C.

DNA qPCR was conducted using TaqMan Universal PCR Master Mix (Applied Biosystems 4305719, Beverly, MA, USA) to quantify total HIV-1 DNA. HIV-1 DNA and approximate cell counts were determined by pNL43 standard curve (2.52–50,000 copies) with primer and probe targeting HIV-1 5′ LTR (see above). PCR conditions were 10 min at 95 °C, 40 cycles of 15 s at 95 °C, and 60 s at 60 °C. Quantified HIV-1 RNA was normalized to HIV-1 DNA.

### 2.8. Chromatin Immunoprecipitation Assay

Approximately 1.5 million HEK 293Ts were infected with NL43-denv-luciferase at MOI of 0.1 24 h prior to transfection with dCas9-KRAB and indicated gRNAs. Forty-eight h post-transfection, cells were crosslinked using 1% paraformaldehyde solution and the reaction was quenched with glycine at the final concentration of 240 mM. Cells were washed and lysed by resuspending in cell lysis buffer (5 mM Tris-HCl pH 8, 90 mM KCl, H2O, 1% NP40 (or IGEPAL), 1× protease inhibitor cocktail) for 10 min at 4 °C followed by nuclear lysis buffer (50 mM Tris-HCl pH 8, 10 mM EDTA, 0.5% SDS, H2O, 25 mM Sodium butyrate, 1× protease inhibitor cocktail) at 4 °C for 15 min. Cell lysates were sonicated in Diagenode Bioruptor Pico (Diagenode Inc, Glenville, NJ, USA) for 15 cycles, 30 s on and 30 s off. Following sonication, cell pellets were resuspended in ‘’RIPA-like’’ buffer (20 mM Tris-HCl pH8, 2 mM EDTA, 0.5 mM EGTA pH 8, 1% Triton X, 140 mM NaCl, H2O, 0.25% sodium deoxycholate, 1× protease inhibitor cocktail) and rocked at 4 °C for 15 min, and chromatin fraction was collected from the supernatant. Chromatin fraction was precleared using 50% protein A sepharose beads and rocked for 30 min at 4 °C. Chromatin fractions containing supernatant were collected and diluted using ChIP dilution buffer 10 mM Tris-HCl pH 8.0, 1 mM EDTA, 0.5 mM EGTA, 0.1% SDS, 1% Triton X, H2O, 0.5 mM PMSF). Antibodies to H3K9me3 (Invitrogen 701784), and acetylated H3 (Millipore Sigma 067–55, Burlington, MA, USA), were added to the diluted chromatin fraction and rotated at 4 °C overnight. Protein A sepharose beads were added and rocked for 2 h at 4 C, followed by two washes each with low salt buffer (0.1% SDS, 1% Triton X-100, 2 mM EDTA, 20 mM Tris-HCl, pH 8, 150 mM NaCl in H2O), high salt buffer (0.1% SDS, 1% Triton X-100, 2 mM EDTA, 20 mM Tris-HCl, pH 8, 500 mM NaCl in H2O), lithium wash buffer (0.25 M LiCl, 1% NP-40, 1% sodium deoxycholate, 1mM EDTA, 10mM Tris-HCl pH 8 in H2O), and TE buffer (10mM Tris-HCl pH 8, 1 mM EDTA, H2O). Protein-DNA complexes were eluted with 10% SDS and treated with proteinase K at 55 °C for 2 h prior to incubating at 65 °C overnight for reverse cross-linking. DNA was extracted using Zymo Research ChIP DNA Clean and Concentrator kit (Zymo Research D5201, D5205, Irvine, CA, USA). Real time qPCR was performed using GoTaq reagents (Promega, Madison WI, USA) with cycling conditions of 94 °C for 4 min, followed by 50 cycles of 94 °C for 15 s, 60 °C for 30 s, and 72 °C for 30 s. Primers targeting +30F/+134R relative to the +1 mRNA transcriptional start site (+30F 5′-CTGGGAGCTCTCTGGCTAACTA-3′; +134R5′-TTACCAGAGTCACACAACA GACG-3′) were used for detection of HIV DNA.

### 2.9. Statistical Analysis

Statistical analyses were performed using GraphPad Prism (GraphPad Prism v 8.4.3 for windows, GraphPad Software, San Diego, California USA, www.graphpad.com). Student *t*-tests were performed. Statistical significance was considered to be *p* ≤ 0.05.

## 3. Results

### 3.1. Repressing HIV Transcription with dCas9-KRAB

**Figure 1:**
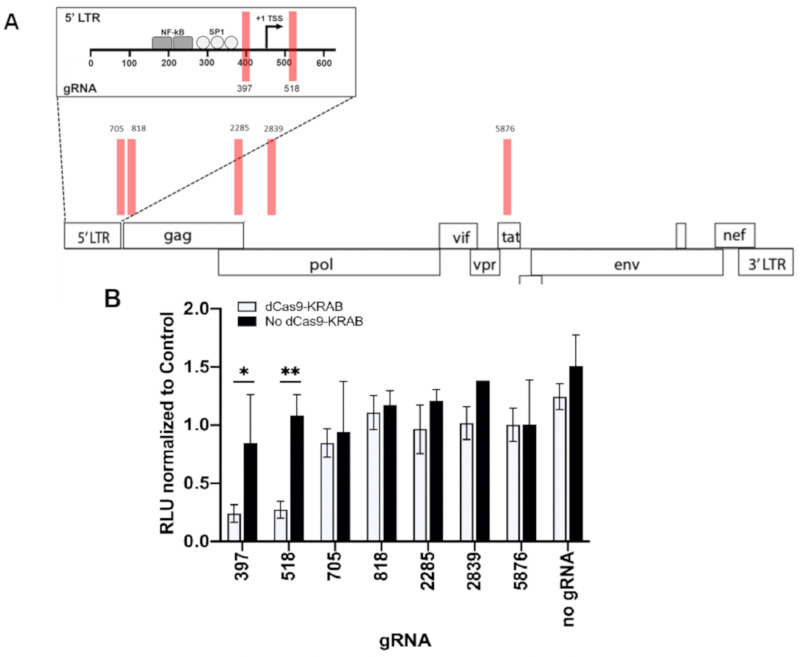
Identification of HIV-1 guide RNAs (gRNAs) that mediate disabled Cas9 (dCas9)- Kruppel-associated box (KRAB) repression. (**A**) Cartoon demonstrating locations targeted by gRNAs. (**B**) Repression of HIV transcription by gRNAs. HEK293T cells were cotransfected with NL4-3-Δ*env*-Luciferase, dCas9-KRAB, and the indicated guide RNAs. Negative controls included transfections with gRNAs only (black bars). HIV expression was monitored after 48 h by luciferase activity, which was normalized to control gRNA. Data represent averages of multiple experiments, with each guide being assessed in at least three independent experiments. Error bars represent standard error and Student *t*-test were performed to determine significance. * and ** indicate *p* ≤ 0.05 and *p* ≤ 0.01, respectively.

We wanted to explore mechanisms by which dCas9-KRAB inhibits HIV-1 transcription. We cotransfected HEK293T cells with NL43-∆*env*-Luciferase and dCas9-KRAB chimeric expression constructs plus candidate gRNAs to identify repressive targets. Guides 397 and 518, which flank the transcriptional start site (TSS) in the LTR, repressed HIV-luciferase expression by greater than 70%, compared to scrambled control and gRNAs targeting regions outside of LTR (Figure 1). Transfection of gRNAs alone did not significantly influence HIV-luciferase expression.

**Figure 2:**
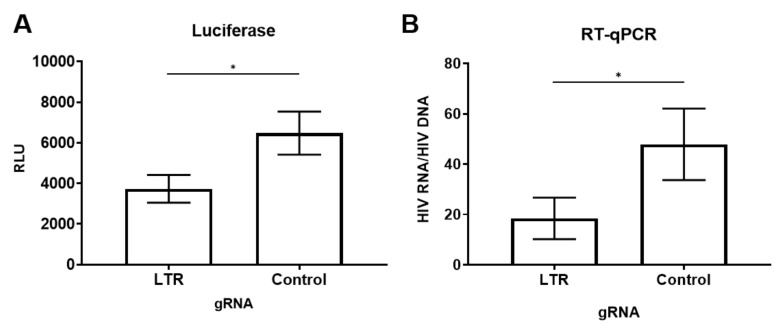
Repression of Proviral HIV transcription. HEK293T cells were infected with NL4-3-env-luciferase (MOI of 0.1) for 24 h and cotransfected with dCas9-KRAB and LTR targeting guide (397) or a scrambled control. Forty-eight hours post transfection, cells were monitored for HIV-1 expression by measuring (**A**) luciferase (*n* = 16 independent experiments) and (**B**) HIV-1 RNA (*n* = 5 independent experiments). Bars represent the mean of these results and error bars are the standard error. Student *t*-test was performed. * indicates *p* ≤ 0.01.

To determine whether dCas9-KRAB and gRNA repressed HIV expression in infected cells, HEK 293T cells were infected with VSVg pseudotyped NL4-3-∆*env*-Luciferase and transfected with dCas9-KRAB and respective gRNAs. Compared to the control gRNA, LTR targeting gRNA resulted in an approximately 50% decrease in luciferase expression (Figure 2A). Combining gRNAs did not enhance repression. Diminished HIV-1 proviral transcription was confirmed by RT-qPCR. HIV RNA transcription per HIV DNA template was decreased by 50% to 90% with gRNA targeting the LTR, compared to the control gRNA (Figure 2B). Proviral HIV-1 transcription was monitored over time by assessing luciferase and RNA expression. Proviral transcription was repressed in infected HEK293T cells for at least 5 days and up to 14 days, suggesting that dCas9-KRAB and guide RNAs facilitated a persistent decrease in HIV transcription (Figure S1). These results demonstrate the ability to actively repress HIV-1 proviral transcription with dCas9-KRAB.

### 3.2. dCas9-KRAB Inhibits Reactivation of Latent HIV

**Figure 3:**
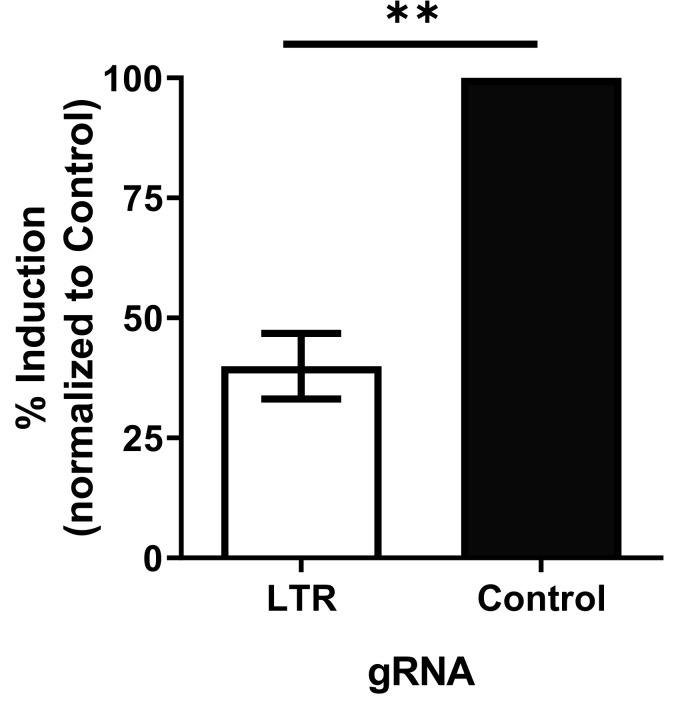
dCas9-KRAB inhibits reactivation of latent proviral HIV. J-Lat 6.3 cells that stably express dsaCas9-KRAB were electroporated with short RNA gRNA targeting LTR (397) or scrambled control. Cells were activated with PMA + Ionomycin, and HIV expression was monitored by qRT-PCR. These data include results from five independent experiments. Error bars represent standard error, and Student t-test was performed to determine significance. ** indicates *p* ≤ 0.01.

We examined if dCas9-KRAB inhibited reactivation of transcriptionally repressed latent provirus. For this experiment, we electroporated J-Lat 6.3 cells that stably express dCas9-KRAB with gRNAs. J-Lat 6.3 are a clonally selected cell line that contains a single copy of HIV-1 with GFP inserted in *nef* and has low basal HIV expression, which is induced in response to T cell activation and latency reversing agents [43]. J-Lat-dCas9-KRAB cells electroporated with specific gRNAs were stimulated with PMA plus ionomycin, and HIV-1 transcription was assessed by RT-qPCR to measure HIV-1 mRNA. RNA expression in which the LTR specific gRNA prevents induction of transcription compared to control gRNA was reduced by 60% (Figure 3). Blocking of reactivation of HIV transcription indicates that dCas9-KRAB prevents reactivation and maintains repression of latent HIV-1 provirus.

### 3.3. dCas9-KRAB Repression of HIV-1 Transcription Correlates with Epigenetic Modifications

The KRAB domain has been shown to function as a transcriptional repressor through its ability to interact with co-repressors, such as KAP-1, which in turn recruits chromatin modifiers like histone deacetylases and histone methyltransferases [49,50,51,52]. We hypothesized that dCas9-KRAB specifically represses transcription by changing the chromatin landscape at the HIV-1 LTR.

**Figure 4:**
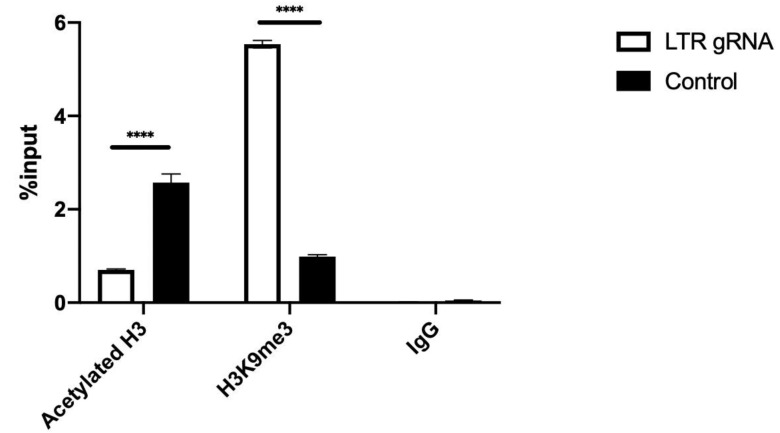
dCas9-KRAB mediates post-translational modification of histones that correlate with transcriptional repression. HEK 293T cells were infected with pseudotyped NL4-3-env-Luciferase and 24 hours later transfected in duplicate with dCas9-KRAB construct and gRNA targeting the HIV LTR (397) or a scrambled control. Forty-eight hours post transfection, ChIPs were performed using anti-acH3 and anti-H3K9me3 antibodies. Associated HIV-1 LTR sequences were detected by PCR. These data represent a single experiment of 3 independent experiments (see Figure S2). Error bars represent standard deviation, and a student *t*-test was performed for statistical significance. **** indicates *p* < 0.001.

We performed ChIP-qPCR on chromatin prepared from HEK293T cells infected with HIV-1 and transfected with dCas9-KRAB and gRNAs. Protein-DNA complexes from infected cells were precipitated with antibodies against acetylated histone3, a histone mark that correlates with active transcription, and H3K9me3, a histone mark that correlates with transcriptional repression. ChIPs show that cells harboring dCas9-KRAB and specific gRNAs targeting the 5′ LTR of HIV-1 had approximately 50% decrease in acetylated histone-3 at +30/+134 base pairs where the repressive nuc-1 is located [53], compared to control gRNAs (Figure 4 and Figure S2). Consistent with dCas9-KRAB facilitating repressive epigenetic changes at the 5′ LTR, we observed three- to five-fold higher levels of H3K9me3 at nuc-1 when transfected with LTR gRNA compared to control gRNA (Figure 4 and Figure S2). As acetylated Histone 3 is a mark of open chromatin and H3K9me3 is a mark of repressed or closed chromatin, these data indicate that dCas9-KRAB represses transcription by recruiting epigenetic modifiers such as histone deacetylases and histone methyltransferases.

## 4. Discussion and Conclusions

The presence of latent but replication-competent HIV-1 provirus in long-lived resting and memory CD4+ T cell populations is a major barrier to curing HIV/AIDS. Strategies to purge or shock and kill latent provirus include inhibitors of histone acetyltransferases, methyltransferase, Bromodomain (BRD) factors, and T-cell-signaling agonist, as well as engineering transcriptional activators, including using chimeric CRISPR/Cas9 or ZnF transcriptional activators [36,37,54]. One drawback to these approaches is that only a fraction of the HIV-1 provirus is transcriptionally induced or is accessible to DNA-modifying agents. CRISPR/Cas9 and targeted endonucleases have been successfully used to excise HIV-1 provirus in vitro [32,33,34] and in animal models [31,35]. Efforts to repress or maintain a transcriptionally silent HIV-1 provirus have also been explored. Block and lock strategies include using molecules that sequester the HIV transcriptional activator Tat, including inhibitor RNAs [55,56] and didehydro-cortistatin A (dCA) [57]. We demonstrate that a chimeric protein that includes dCas9 fused to the KRAB repression domain inhibits HIV-1 transcription and prevents the induction of latent provirus through epigenetic-mediated mechanisms that target the LTR.

We show that targeting dCas9-KRAB to the 5′ LTR of HIV-1 results in robust repression of HIV transcription, compared to targeting other regions of HIV-1 genome. These results are consistent with recent findings showing that repurposed CRISPR/Cas9 fused to transcriptional activators targeted to the 5′ LTR reverse HIV latency in vitro [36,37,54]. Furthermore, our results demonstrate that dCas9-KRAB are facilitating post-translational histone modifications at the LTR, including increased levels of H3K9me3 and decreased levels of acetylated H3, compared to control cells consistent with epigenetic changes associated with HIV-1 latency. KRAB recruits the cofactor KAP1 (also known as TRIM 28 and TIF1b) to promote durable heritable epigenetic chromatin modifications, and has been proposed to maintain heterochromatin and repress transcription of endogenous retroelements [38,39,40,41,42,58,59]. It should be noted that KAP1 activity may be context-dependent, and has been suggested to be an activator as well as a repressor for HIV-1 transcription [60].

In summary, we demonstrate a proof of concept that CRISPR technologies can be repurposed to directly target HIV-1 provirus to repress proviral transcription by promoting repressive epigenetic modifications. These studies provide an additional tool to extinguish HIV-1 proviral expression and suggest the potential of using engineered targeting factors as a strategy for long-term repression and permanent inactivation of the HIV-1 provirus.

## Figures and Tables

**Table 1:** Guide RNA (gRNA) sequence characteristics.

gRNA	Gene	Strand	Sequence (5′-3′)	PAM	Specificity ^a^	Activity ^b^
397	5′ LTR	+	GGTGTGGCCTGGGCGGGACTG	GGGAGT	67.2	6.2
518	5′ LTR	−	AGCTTTATTGAGGCTTAAGCA	GTGGGT	83.2	61.6
705	NCR	−	TGCCGTGCGCGCTTCAGCAAG	CCGAGT	89.6	27.4
818	gag	+	GAGCGTCAGTATTAAGCGGGG	GAGAAT	97.1	22.8
2285	gag, pol	−	CCCCCTATCTTTACTTTGACG	AGGGGT	91.0	27.5
2839	pol	+	AATTAGGAATACCACATCCCG	CAGGGT	96.4	66.4
5876	tat	−	AAGCAGTTTTAGGCTGACTTC	CTGGAT	79.5	3.1

^a^ Specificity scores (01–00) are derived to determine off-target binding of gRNA to reference human genome. Higher scores indicate greater specificity for sequence of interest [44,46]. ^b^ Activity scores (01–00) are predicted endonuclease activity at the target site based on gRNA secondary structures. Higher scores indicate greater activity [44,45].

**Table 2:** Disabled Cas9 (dCas9) constructs.

Construct	Type	Description
dCas9-KRAB	Plasmid	pCMV-dsaCas9-KRAB
dCas9-KRAB-Blasticidin^R^	Plasmid	pHR-SFFV-KRAB-dsaCas9-T2A-Blast^R^; stable J-Lat 6.3
gRNA Control	Plasmid	phU6-gRNA (gRNA scaffold)
gRNA 397	Plasmid	phU6-gRNA_Nl43-397
gRNA 518	Plasmid	phU6-gRNA_NL43-518
gRNA 705	Plasmid	phU6-gRNA_NL43-705
gRNA 818	Plasmid	phU6-gRNA_NL43-818
gRNA 2285	Plasmid	phU6-gRNA_NL43-2285
gRNA 2839	Plasmid	phU6-gRNA_NL43-2839
gRNA 5876	Plasmid	phU6-gRNA_NL43-5876
gRNA 397	Ultramer Oligo RNA	−
gRNA Scramble	Ultramer Oligo RNA	−

## Supplementary Materials

The following are available online at https://www.mdpi.com/1999-4915/12/10/1154/s1, Figure S1: Repression of HIV-1 transcription over time by dCas9-KRAB. Figure S2: dCas9-KRAB mediates post-translational modification of histones at the HIV-1 LTR.

## References

[1] Finzi D., Blankson J., Siliciano J.D., Margolick J.B., Chadwick K., Pierson T., Smith K., Lisziewicz J., Lori F., Flexner C. (1999). Latent infection of CD4+ T cells provides a mechanism for lifelong persistence of HIV-1, even in patients on effective combination therapy. Nat. Med..

[2] Siliciano J.D., Kajdas J., Finzi D., Quinn T.C., Chadwick K., Margolick J.B., Kovacs C., Gange S.J., Siliciano R.F. (2003). Long-term follow-up studies confirm the stability of the latent reservoir for HIV-1 in resting CD4+ T cells. Nat. Med..

[3] Abrahams M.R., Joseph S.B., Garrett N., Tyers L., Moeser M., Archin N., Council O.D., Matten D., Zhou S., Doolabh D. (2019). The replication-competent HIV-1 latent reservoir is primarily established near the time of therapy initiation. Sci. Transl. Med..

[4] Chun T.W., Carruth L., Finzi D., Shen X., DiGiuseppe J.A., Taylor H., Hermankova M., Chadwick K., Margolick J., Quinn T.C. (1997). Quantification of latent tissue reservoirs and total body viral load in HIV-1 infection. Nat. Cell Biol..

[5] Wong J.K., Hezareh M., Günthard H.F., Havlir D.V., Ignacio C.C., Spina C.A., Richman D.D. (1997). Recovery of Replication-Competent HIV Despite Prolonged Suppression of Plasma Viremia. Science.

[6] Chavez L., Calvanese V., Verdin E. (2015). HIV Latency Is Established Directly and Early in Both Resting and Activated Primary CD4 T Cells. PLoS Pathog..

[7] Agosto L.M., Herring M.B., Mothes W., Henderson A.J. (2018). HIV-1-Infected CD4+ T Cells Facilitate Latent Infection of Resting CD4+ T Cells through Cell-Cell Contact. Cell Rep..

[8] Spina C.A., Guatelli J.C., Richman D.D. (1995). Establishment of a stable, inducible form of human immunodeficiency virus type 1 DNA in quiescent CD4 lymphocytes in vitro. J. Virol..

[9] Gagne M., Michaels D., Lester G.M.S., Gummuluru S., Wong W.W., Henderson A.J. (2019). Strength of T cell signaling regulates HIV-1 replication and establishment of latency. PLoS Pathog..

[10] Oswald-Richter K., Grill S.M., Leelawong M., Unutmaz D. (2004). HIV infection of primary human T cells is determined by tunable thresholds of T cell activation. Eur. J. Immunol..

[11] Shan L., Deng K., Gao H., Xing S., Capoferri A.A., Durand C.M., Rabi S.A., Laird G.M., Kim M., Hosmane N.N. (2017). Transcriptional Reprogramming during Effector-to-Memory Transition Renders CD4+ T Cells Permissive for Latent HIV-1 Infection. Immunity.

[12] Buzon M.J., Sun H., Li C., Shaw A., Seiss K., Ouyang Z., Martin-Gayo E., Leng J., Henrich T.J., Li J.Z. (2014). HIV-1 persistence in CD4+ T cells with stem cell–like properties. Nat. Med..

[13] Couturier J., Orozco A.F., Liu H., Budhiraja S., Siwak E.B., Nehete P.N., Sastry K.J., Rice A.S.C., Lewis D.E. (2019). Regulation of cyclin T1 during HIV replication and latency establishment in human memory CD4 T cells. Virol. J..

[14] Dobrowolski C., Valadkhan S., Graham A.C., Shukla M., Ciuffi A., Telenti A., Karn J., Ott M., Henderson A., Spina C.A. (2019). Entry of Polarized Effector Cells into Quiescence Forces HIV Latency. mBio.

[15] Tyagi M., Pearson R.J., Karn J. (2010). Establishment of HIV Latency in Primary CD4+ Cells Is due to Epigenetic Transcriptional Silencing and P-TEFb Restriction. J. Virol..

[16] Margolis D.M., Archin N.M., Cohen M.S., Eron J.J., Ferrari G., Garcia J.V., Gay C.L., Goonetilleke N., Joseph S.B., Swanstrom R. (2020). Curing HIV: Seeking to Target and Clear Persistent Infection. Cell.

[17] Martin A.R., Siliciano R.F. (2016). Progress Toward HIV Eradication: Case Reports, Current Efforts, and the Challenges Associated with Cure. Annu. Rev. Med..

[18] Schwarzer R., Gramatica A., Greene W.C. (2020). Reduce and Control: A Combinatorial Strategy for Achieving Sustained HIV Remissions in the Absence of Antiretroviral Therapy. Viruses.

[19] Spivak A.M., Planelles V. (2016). HIV-1 Eradication: Early Trials (and Tribulations). Trends Mol. Med..

[20] Nixon C.C., Mavigner M., Sampey G.C., Brooks A.D., Spagnuolo R.A., Irlbeck D.M., Mattingly C., Ho P.T., Schoof N., Cammon C.G. (2020). Systemic HIV and SIV latency reversal via non-canonical NF-κB signalling in vivo. Nat. Cell Biol..

[21] Abner E., Jordan A. (2019). HIV “shock and kill” therapy: In need of revision. Antivir. Res..

[22] Olson A., Basukala B., Wong W.W., Henderson A.J. (2019). Targeting HIV-1 proviral transcription. Curr. Opin. Virol..

[23] VanSant G., Bruggemans A., Janssens J., Debyser Z. (2020). Block-And-Lock Strategies to Cure HIV Infection. Viruses.

[24] Benjamin R., Berges B.K., Solis-Leal A., Igbinedion O., Strong C.L., Schiller M.R. (2016). TALEN gene editing takes aim on HIV. Qual. Life Res..

[25] Gaj T., Gersbach C.A., Barbas C.F. (2013). ZFN, TALEN, and CRISPR/Cas-based methods for genome engineering. Trends Biotechnol..

[26] Kwarteng A., Ahuno S., Kwakye-Nuako G. (2017). The therapeutic landscape of HIV-1 via genome editing. AIDS Res. Ther..

[27] Kang H., Minder P., Park M.A., Mesquitta W.T., Torbett B., Slukvin I.I. (2015). CCR5 Disruption in Induced Pluripotent Stem Cells Using CRISPR/Cas9 Provides Selective Resistance of Immune Cells to CCR5-tropic HIV-1 Virus. Mol. Ther. Nucleic Acids.

[28] Liu Z., Chen S., Jin X., Wang Q., Yang K., Li C., Xiao Q., Hou P., Liu S., Wu S. (2017). Genome editing of the HIV co-receptors CCR5 and CXCR4 by CRISPR-Cas9 protects CD4+ T cells from HIV-1 infection. Cell Biosci..

[29] Teque F., Ye L., Xie F., Wang J., Morvan M.G., Kan Y.W., Levy J.A. (2020). Genetically-edited induced pluripotent stem cells derived from HIV-1-infected patients on therapy can give rise to immune cells resistant to HIV-1 infection. AIDS.

[30] Yu S., Yao Y., Xiao H., Li J., Liu Q., Yang Y., Adah D., Lu J., Zhao S., Qin L. (2018). Simultaneous Knockout of CXCR4 and CCR5 Genes in CD4+ T Cells via CRISPR/Cas9 Confers Resistance to Both X4- and R5-Tropic Human Immunodeficiency Virus Type 1 Infection. Hum. Gene Ther..

[31] Dash P.K., Kaminski R., Bella R., Su H., Mathews S., Ahooyi T.M., Chen C., Mancuso P., Sariyer R., Ferrante P. (2019). Sequential LASER ART and CRISPR Treatments Eliminate HIV-1 in a Subset of Infected Humanized Mice. Nat. Commun..

[32] Ebina H., Misawa N., Kanemura Y., Koyanagi Y. (2013). Harnessing the CRISPR/Cas9 system to disrupt latent HIV-1 provirus. Sci. Rep..

[33] Hu W., Kaminski R., Yang F., Zhang Y., Cosentino L., Li F., Luo B., Alvarez-Carbonell D., Garcia-Mesa Y., Karn J. (2014). RNA-directed gene editing specifically eradicates latent and prevents new HIV-1 infection. Proc. Natl. Acad. Sci. USA.

[34] Park R.J., Wang T., Koundakjian D., Hultquist J.F., Lamothe-Molina P., Monel B., Schumann K., Yu H., Krupzcak K.M., Garcia-Beltran W. (2016). A genome-wide CRISPR screen identifies a restricted set of HIV host dependency factors. Nat. Genet..

[35] Yin C., Zhang T., Qu X., Zhang Y., Putatunda R., Xiao X., Li F., Xiao W., Zhao H., Dai S. (2017). In Vivo Excision of HIV-1 Provirus by saCas9 and Multiplex Single-Guide RNAs in Animal Models. Mol. Ther..

[36] Bialek J.K., Dunay G.A., Voges M., Schäfer C., Spohn M., Stucka R., Hauber J., Lange U.C. (2016). Targeted HIV-1 Latency Reversal Using CRISPR/Cas9-Derived Transcriptional Activator Systems. PLoS ONE.

[37] Limsirichai P., Gaj T., Schaffer D. (2016). CRISPR-mediated Activation of Latent HIV-1 Expression. Mol. Ther..

[38] Azzaz A.M., Vitalini M.W., Thomas A.S., Price J.P., Blacketer M.J., Cryderman D.E., Zirbel L.N., Woodcock C.L., Elcock A.H., Wallrath L.L. (2014). Human Heterochromatin Protein 1α Promotes Nucleosome Associations That Drive Chromatin Condensation. J. Biol. Chem..

[39] Iyengar S., Farnham P.J. (2011). KAP1 Protein: An Enigmatic Master Regulator of the Genome. J. Biol. Chem..

[40] Ma X., Yang T., Luo Y., Wu L., Jiang Y., Song Z., Pan T., Liu B., Liu G., Liu J. (2019). TRIM28 promotes HIV-1 latency by SUMOylating CDK9 and inhibiting P-TEFb. eLife.

[41] Meylan S., Groner A.C., Ambrosini G., Malani N., Quenneville S., Zangger N., Kapopoulou A., Kauzlaric A., Rougemont J., Ciuffi A. (2011). A gene-rich, transcriptionally active environment and the pre-deposition of repressive marks are predictive of susceptibility to KRAB/KAP1-mediated silencing. BMC Genom..

[42] Yang B.X., El Farran C.A., Guo H.C., Yu T., Fang H.T., Wang H.F., Schlesinger S., Seah Y.F.S., Goh G.Y.L., Neo S.P. (2015). Systematic identification of factors for provirus silencing in embryonic stem cells. Cell.

[43] Jordan A., Bisgrove D., Verdin E. (2003). HIV reproducibly establishes a latent infection after acute infection of T cells in vitro. EMBO J..

[44] Benchling. http://www.benchling.com.

[45] Doench J.G., Fusi N., Sullender M., Hegde M., Vaimberg E.W., Donovan K.F., Smith I., Tothova Z., Wilen C., Orchard R. (2016). Optimized sgRNA design to maximize activity and minimize off-target effects of CRISPR-Cas9. Nat. Biotechnol..

[46] Hsu P.D., Scott D., Weinstein J., Ran F.A., Konermann S., Agarwala V., Li Y., Fine E.J., Wu X., Shalem O. (2013). DNA targeting specificity of RNA-guided Cas9 nucleases. Nat. Biotechnol..

[47] Kiani S., Chavez A., Tuttle M., Hall R.N., Chari R., Ter-Ovanesyan D., Qian J., Pruitt B.W., Beal J., Vora S. (2015). Cas9 gRNA engineering for genome editing, activation and repression. Nat. Methods.

[48] Bintu L., Yong J., Antebi Y., McCue K., Kazuki Y., Uno N., Oshimura M., Elowitz M.B. (2016). Dynamics of epigenetic regulation at the single-cell level. Science.

[49] Peng H., Begg G., Schultz D.C., Friedman J.R., Jensen D., Speicher D.W., Rauscher F.J. (2000). Reconstitution of the KRAB-KAP-1 repressor complex: a model system for defining the molecular anatomy of RING-B box-coiled-coil domain-mediated protein-protein interactions. J. Mol. Biol..

[50] Urrutia R. (2003). KRAB-containing zinc-finger repressor proteins. Genome Biol..

[51] Schultz D.C. (2002). SETDB1: A novel KAP-1-associated histone H3, lysine 9-specific methyltransferase that contributes to HP1-mediated silencing of euchromatic genes by KRAB zinc-finger proteins. Genes Dev..

[52] Sripathy S.P., Stevens J., Schultz D.C. (2006). The KAP1 Corepressor Functions To Coordinate the Assembly of De Novo HP1-Demarcated Microenvironments of Heterochromatin Required for KRAB Zinc Finger Protein-Mediated Transcriptional Repression. Mol. Cell. Biol..

[53] Agosto L.M., Gagne M., Henderson A.J. (2015). Impact of Chromatin on HIV Replication. Genes.

[54] Saayman S.M., Lazar D.C., Scott T.A., Hart J.R., Takahashi M., Burnett J.C., Planelles V., Morris K.V., Weinberg M.S. (2016). Potent and Targeted Activation of Latent HIV-1 Using the CRISPR/dCas9 Activator Complex. Mol. Ther..

[55] Jin H., Li D., Lin M.H., Li L., Harrich D. (2020). Tat-Based Therapies as an Adjuvant for an HIV-1 Functional Cure. Viruses.

[56] Mousseau G., Valente S.T. (2017). Role of Host Factors on the Regulation of Tat-Mediated HIV-1 Transcription. Curr. Pharm. Des..

[57] Mousseau G., Clementz M.A., Bakeman W.N., Nagarsheth N., Cameron M., Shi J., Baran P., Fromentin R., Chomont N., Valente S.T. (2012). An Analog of the Natural Steroidal Alkaloid Cortistatin A Potently Suppresses Tat-Dependent HIV Transcription. Cell Host Microbe.

[58] Friedli M., Trono D. (2015). The Developmental Control of Transposable Elements and the Evolution of Higher Species. Annu. Rev. Cell Dev. Biol..

[59] Wolf G., Greenberg D., Macfarlan T.S. (2015). Spotting the enemy within: Targeted silencing of foreign DNA in mammalian genomes by the Krüppel-associated box zinc finger protein family. Mob. DNA.

[60] Morton E.L., Forst C.V., Zheng Y., DePaula-Silva A.B., Ramirez N.G.P., Planelles V., D’Orso I. (2019). Transcriptional Circuit Fragility Influences HIV Proviral Fate. Cell Rep..

