# Supplementary material for: Targeted Chromatinization and Repression of HIV-1 Provirus Transcription with Repurposed CRISPR/Cas9
Source: Viruses. 2020 Oct 12;12(10):1154. doi: 10.3390/v12101154 (PMC7600714; doi:10.3390/v12101154)
Supplement: Supplementary file 1 [file viruses-12-01154-s001.pdf]

SUPPLEMENTAL FIGURES

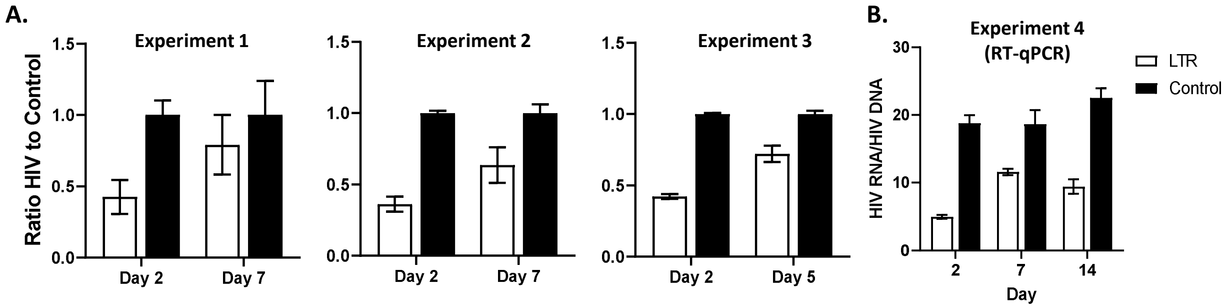

Supplementary Figure 1. HEK293T cells were infected with NL4-3-env-Luciferase (MOI of 0.1) for 24 h and cotransfected with dCas9-KRAB and LTR targeting guide (397) or a scrambled control. Cells were monitored for HIV-1 expression by measuring luciferase on the indicated days post-transfection. Each panel represents an individual experiment performed in triplicate. A) RLU normalized to control. Error bars represent standard deviation. B) HIV-1 RNA per HIV-1 DNA as determined by RT-qPCR from a single experiment performed with duplicate samples. Error bars represent standard deviation.

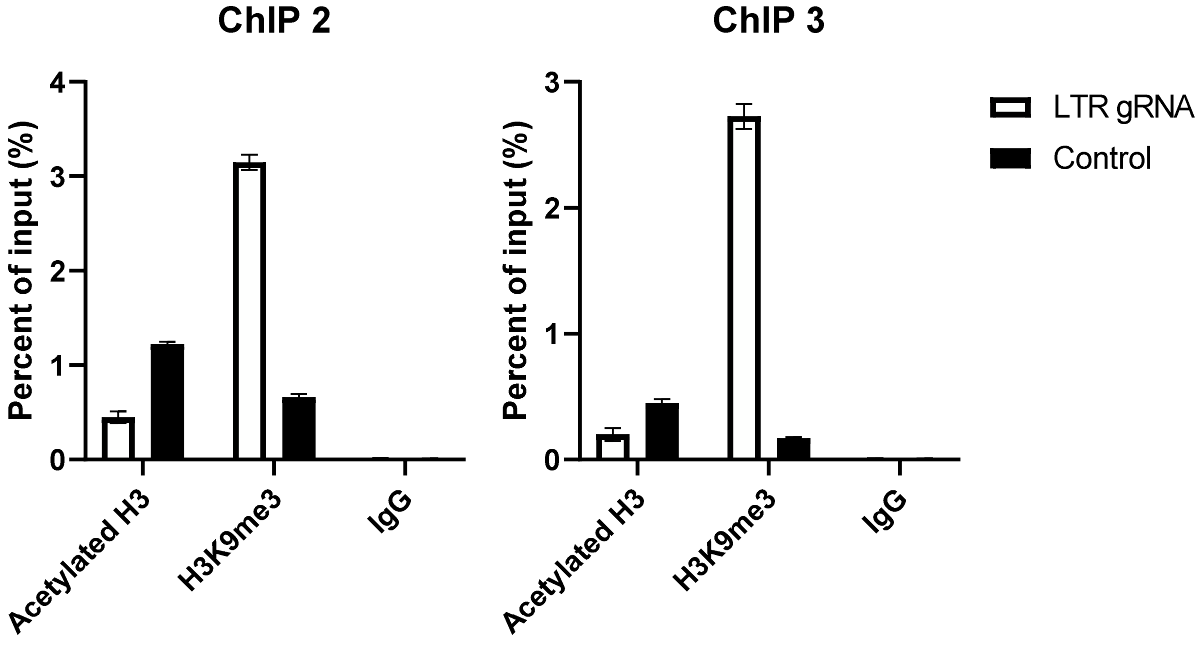

Supplementary Figure 2. HEK 293T cells were infected with pseudotyped NL4-3-env-Luciferase and 24 hours later transfected with dCas9-KRAB construct and gRNA targeting the HIV LTR (397) or a scrambled control. Forty-eight hours post transfection, cells were collected, and ChIPs were performed using anti-acH3 and anti-H3K9me3 antibodies. Associated HIV-1 LTR sequences were detected by PCR. Each panel is a single experiment performed in triplicate and error bars represent standard deviation.
